# Understanding the qPCR Standard Curve: From Assay Validation to Absolute Quantification and Variance PCR

**DOI:** 10.3390/ijms27062904

**Published:** 2026-03-23

**Authors:** Mikael Kubista, Amin Forootan, Michael W. Pfaffl, Stephen A. Bustin, Jose M. Andrade, Robert Sjöback, Björn Sjögreen, Anders Ståhlberg

**Affiliations:** 1 Institute of Biotechnology, Czech Academy of Science, 252 42 Vestec, Czech Republic; 2 Sahlgrenska Center for Cancer Research, Department of Laboratory Medicine, Institute of Biomedicine, Sahlgrenska Academy, University of Gothenburg, 413 90 Gothenburg, Sweden; anders.stahlberg@gu.se; 3 MultiD Analyses AB, GoCo Health Innovation City, 431 53 Mölndal, Sweden; amin.forootan@multid.se (A.F.); bjorn.sjogreen@multid.se (B.S.); 4 Animal Physiology and Immunology, School of Life Sciences, Technical University of Munich, 85354 Freising, Germany; michael.pfaffl@tum.de; 5 Medical Technology Research Centre, Faculty of Health, Education, Medicine and Social Care, Anglia Ruskin University, Chelmsford CM1 1SQ, UK; stephen.bustin@aru.ac.uk; 6 Group of Applied Analytical Chemistry, University of A Coruña, Campus da Zapateira, 15008 A Coruña, Spain; jose.manuel.andrade@udc.es; 7 Precision BioAnalytics AB, GoCo Health Innovation City, 431 53 Mölndal, Sweden; robert.sjoback@precisionbioanalytics.com; 8 Wallenberg Centre for Molecular and Translational Medicine, University of Gothenburg, 413 90 Gothenburg, Sweden; 9 Department of Clinical Genetics and Genomics, Sahlgrenska University Hospital, 413 45 Gothenburg, Region Västra Götaland, Sweden; 10 Science for Life Laboratory, Institute of Biomedicine, University of Gothenburg, 405 30 Gothenburg, Sweden

**Keywords:** PCR, real-time PCR, qPCR, variation PCR, varPCR, RT-PCR, standard curve, MIQE, molecular diagnostics

## Abstract

The quantitative polymerase chain reaction (PCR) standard curve is the central analytical tool for validating qPCR assays and can also be used to estimate target concentrations in test samples. This review explains how qPCR standard curves are constructed, validated, and analyzed for different purposes. We first examine an idealized standard curve generated using an exceptionally high number of replicates, far exceeding typical routine use. This approach clearly illustrates fundamental qPCR characteristics and provides an educational framework for defining and estimating PCR efficiency, limit of detection, and limit of quantification. Furthermore, we demonstrate that, in theory, variation in threshold crossing points across replicates can be used to estimate the number of target molecules in a sample. This method, which we term variance PCR, could complement digital PCR and potentially extend the dynamic range of absolute quantification. We also analyze a representative standard curve as typically processed in routine qPCR workflows. This includes validating its dynamic range, assessing the impact of outliers, estimating PCR efficiency and precision, and finally applying the curve to determine the concentration of test samples.

## 1. Introduction

Real-time quantitative PCR (qPCR) is arguably the predominant technology for nucleic acid quantification and serves as a benchmark for validating other methods. Reliable qPCR, however, requires rigorous assay validation, as outlined by the ‘Minimum Information for Publication of Quantitative Real-Time PCR Experiments’ (MIQE) guidelines, originally published in 2009 [1], and recently updated [2]. The MIQE guidelines served as the basis for the ISO 20395:2019 standard, “Biotechnology—Requirements for evaluating the performance of quantification methods for nucleic acid target sequences—qPCR and dPCR”, in diagnostics [3], and also for the white paper “Recommendations for Method Development and Validation of qPCR and dPCR Assays in Support of Cell and Gene Therapy Drug Development” [4].

Despite these frameworks, erroneous qPCR results, often arising from flawed experimental design, inadequate validation, and incorrect data analysis, remain common in the literature, sometimes with serious consequences. A recent comprehensive review of qPCR methodological standards and reporting practices has shown that many of these deficiencies are still widespread, underscoring the gap between published guidance and routine practice [5].

The standard curve is the central tool for proper qPCR analysis, serving both to validate assay performance and to quantify target molecules in test samples. This paper aims to clarify the construction, interpretation, and validation of qPCR standard curves, with a particular focus on distinguishing their distinct roles in assay validation versus sample quantification, a distinction that is frequently blurred in practice.

We begin by analyzing a deliberately idealized standard curve constructed with an extreme number of replicates. This didactic example makes fundamental qPCR properties, such as sampling uncertainty, limits of detection and quantification, and the relationship between the variation of the quantification cycle (Cq) across replicate amplification curves, explicitly visible. We then examine standard curves under typical, replication-limited conditions, demonstrating how to evaluate dynamic range, outliers, efficiency, and prediction uncertainty in routine practice.

The insights from the idealized case led to a conceptual extension we term variance PCR (varPCR), which clarifies the theoretical relationship between Cq variation and absolute target copy number. This framework is intended to elucidate the fundamental limits of quantification rather than to propose a new routine method. Throughout, the emphasis is on understanding what a standard curve can and cannot reveal, thereby helping to prevent common interpretative errors.

## 2. Constructing the qPCR Standard Curve

**Figure 1:**
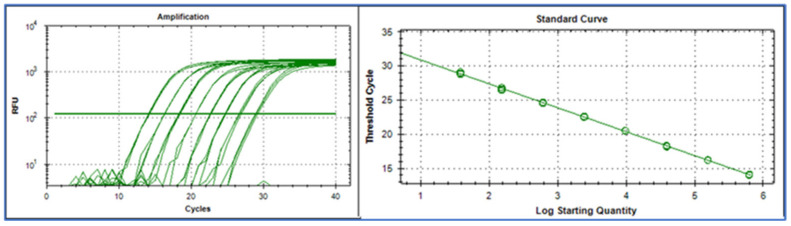
The Cq values, extracted from the amplification curves ((**left**), RFU = relative fluorescence units), are plotted versus the starting amount of target molecules on a logarithmic scale of the standard samples (**right**). Data were generated using a Bio-Rad CFX384 and analyzed with the CFX Manager Software ver. 3.1.

A qPCR standard curve is constructed by plotting the Cq-value against the logarithm of the target amount, expressed as copy number, concentration, or sometimes dilution factor (Figure 1).

The Cq values, known also as ‘cycle threshold’ (Ct), ‘crossing point’ (Cp), or ‘take-off point’ (TOP) in older literature, are extracted from the qPCR amplification curves and reflect the amount of target in the samples. There are several strategies for extracting Cq values. The most common way is to read them out as the crossing points of the amplification curves with a threshold level. Although the choice of the threshold influences the Cq values, as long as the amplification curves are parallel at the threshold level, the relative spacing of the curves is preserved, and comparisons of Cq values across samples remain valid. 

The standard curve data are fitted to a straight line using the linear regression of Equation (1):

(1) Cq(N)=k×log(N)+m

where N reflects the expected number of target molecules, optionally normalized to volume, being a concentration. For samples with high target concentrations, the expected concentration is what we typically refer to as concentration: the average number of target molecules per analyzed volume. However, as we shall discuss, analyzing aliquots (or samplings, as they are also called) that contain just a few target molecules, a variation across them is expected that affects the measured Cq. In this work, we therefore refer to the expected number of target molecules per aliquot as N, while the actual number of target molecules in a particular aliquot is x. The average of x across aliquots should equal N. k is the slope of the standard curve. m is the intercept and corresponds to the Cq of a sample containing a single, in our case, double-stranded target molecule, m = Cq(1) [6].

Alternatively, the Cq values are taken as the second derivative maximum of the amplification curve, which is based on a smoothed sigmoidal or logistic mathematical model. 

A note about jargon is in order here. Following IUPAC, a “sample” is a portion of material selected from a larger quantity of material [7]. The term usually needs qualification (e.g., bulk sample, raw sample, etc.), and it implies the existence of a sampling error, i.e., the results obtained on the portions taken are only estimates of the number of target molecules. If there is no or negligible sampling error, the portion removed is a test portion, aliquot, or specimen [7]. In this paper, we will not deal with sampling variability and just assume that a sample was submitted for analysis at the laboratory. Withdrawal of one or several working sample portions (aliquots, each is considered a replicate) is the current way of working, and so we will focus on them (otherwise explained).

Standard curves serve two distinct purposes: (1) validation of assays, and (2) estimation of target concentrations in test samples by comparison with standards. The design and interpretation of the standard curve differ depending on its intended use.

For every PCR assay, performance assessment is a critical component of assay validation. This should be conducted under optimal conditions, i.e., in the absence of interfering substances, such as inhibitors and enhancers. For most targets, a PCR efficiency of 90–100% should be achievable. 

Calibration samples (the preferred term [7,8]), calibrators (a shortcut for the former term, using the word in a pragmatic loose way [7]), and standards or standard samples (this term is ambiguous) are used for constructing a standard curve; they are typically prepared by serial dilution of a concentrated stock solution with a known target concentration. One or several replicates of each calibration sample can be measured. Of paramount importance is to determine accurately the concentration of this stock solution, which represents a very first challenge because the concentration of synthetic oligonucleotides provided by oligo manufacturers is usually not precise enough for qPCR, and validation is needed.

**Figure 2:**
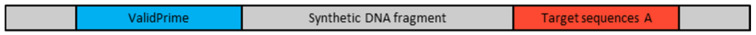
Design of a synthetic reference material comprising a target and a reference sequence in the same DNA molecule, guaranteeing a 1:1 ratio.

A strategy is to design synthetic templates to include both the target sequence and a reference sequence, for which there is a well-characterized, high-performing qPCR assay, in a 1:1 ratio (Figure 2). One such reference sequence and assay for it is discussed below. It was originally designed as a means to assess the genomic DNA background in expression analysis [9] and is one of the most well-characterized qPCR reference assays [10]. The concentration of the stock solution can then be determined with high precision by targeting the reference sequence using digital PCR (dPCR).

Once an optimized qPCR assay has been established, it is advisable to evaluate its performance (this is validation) with a sample matrix, referred to as a blank or procedural blank, that is processed according to the established protocol. The processed matrix is then spiked with reference standard DNA of known concentration, and a new standard curve is generated. PCR efficiency is re-estimated under these conditions to assess the impact of matrix-related interference. Hence, it is worth noting that PCR efficiency is sample-type dependent in the absence of other dominating factors. Optimized assays are generally robust, and matrix effects are typically limited. However, if substantial effects are observed, the protocol may require additional optimization or, if assay robustness is in question, redesign. 

The second application of a standard curve is the quantification of targets in test samples. This is performed by comparison with standard samples (calibrators) of known concentrations within a validated dynamic range. In this case, they should be constructed differently. They should be independent of one another to capture the variability inherent in independent test samples and should reflect as much of the analytical workflow as possible. Ideal standards consist of a naïve sample matrix spiked with a known amount of target. If the target in test samples is expected to be contained or protected, such as a nucleic acid encapsulated in lipid nanoparticles or adeno-associated viruses, the standards should mimic this protection [11]. The calibration samples are then processed identically to the test samples prior to construction of the standard curve.

## 3. Learnings from an Extreme Standard Curve

We begin by examining an extreme standard curve constructed using a very large number of replicates per concentration and small concentration increments. This example is included as a deliberately idealized, illustrative case to highlight fundamental properties of qPCR behavior that are not readily visible in routine standard curves. It represents a validation experiment for the reference sequence (Figure 1) [9] performed using the IntelliQube instrument that uses very small reaction volumes, enabling high throughput [10]. The example data with tutorials is available [12].

The reference sequences were originally proposed as a cost-efficient strategy to correct for background genomic DNA (gDNA) in gene expression analysis. The reference sequences are species-specific, and each targets a conserved, non-transcribed genomic sequence present in exactly one copy per haploid genome. This allows for accurate quantification of the gDNA background in complementary DNA (cDNA) samples [9]. 

Calibration samples were prepared from a stock solution of purified synthetic DNA containing a single reference sequence per double-stranded molecule. The concentration of the stock solution was determined using dPCR with the reference sequence assay. Solutions covering the range of 2048 down to 1 target molecule on average per reaction were prepared using 2-fold dilution steps. At the lowest concentration, and for the non-template control, 128 replicates were assessed. Higher concentrations were replicated 64 times and the highest 32 times.

**Figure 3:**
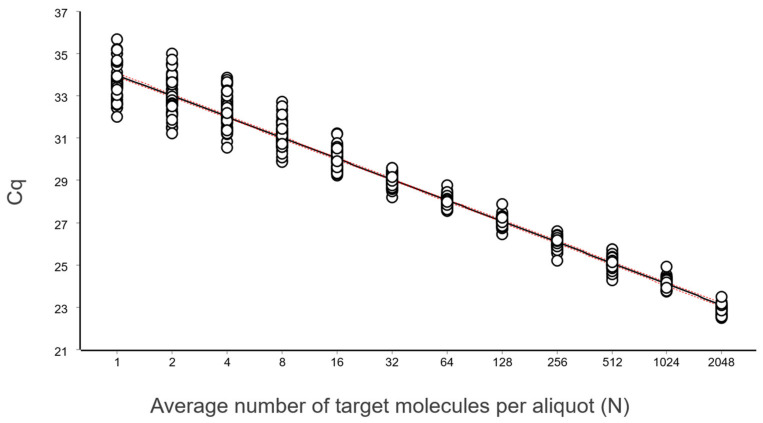
Calibration samples containing on average (N): 1, 2, 4, 8, 16, 32, 64, 128, 256, 512, 1024, and 2048 target molecules per aliquot analyzed in replicates using the reference sequence assay. The number of replicates was: 128 (N = 1), 64 (2 ≤ N ≤ 512), and 32 (1024 ≤ N ≤ 2048). Data fitted to a black straight line with ordinary linear regression. Working–Hotelling confidence area indicated with red dashed lines.

The data, after removing some obviously technically failed reactions, fitted to a straight line using linear regression (ordinary least squares fit, OLS), as shown in Figure 3.

### 3.1. Residual Plot

**Figure 4:**
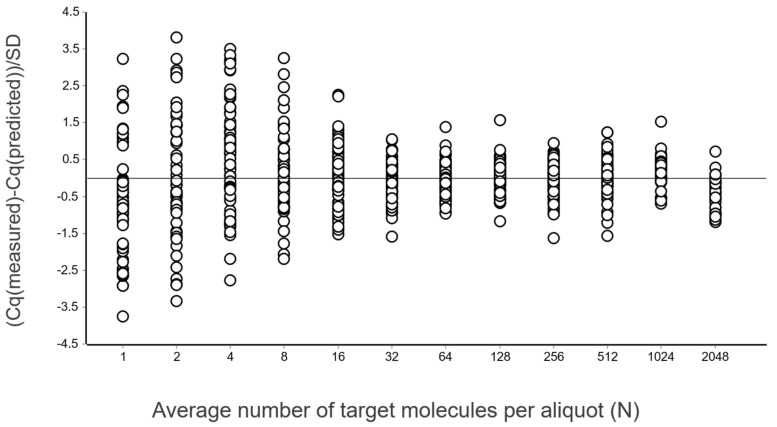
Residual plot of the data in Figure 3 expressed in standard deviations.

A general good practice is to study the differences between Cq values and those predicted by the linear regression, i.e., the residuals. This is done using a residuals plot. Either the combined data at all levels (different concentrations) can be considered, as in Figure 4, or per each level separately [13].

From these illustrative data with a very large number of replicates, we see in Figure 3 and Figure 4 that at lower N, the variation, which can be quantified as the standard deviation (SD), becomes higher. This violates one of the assumptions of the OLS criterion. Either weighted least squares should be applied, or, as we will do below, only samples above the lower limit of quantification (LLOQ) will be considered.

### 3.2. Relative Standard Deviation and Limit of Quantification

The variation across replicates can be quantified by means of the SD calculated for each calibrator, where SD can be calculated either for the measured Cq-values or for concentrations derived from the Cq-values. These SDs are very different because of the difference in scale. While concentrations are in a linear scale, Cq-values are in a log scale. For comparison with other bioanalytical methods, performance parameters are preferably presented on a linear scale.

The SD, like the mean, increases with the expected number of target molecules per sample. For comparison across concentrations, the relative standard deviation (RSD), also referred to as the coefficient of variation, is the most widely used measure of imprecision. In a linear scale, RSD is obtained from the SD of the Cq values as depicted in Equation (2) (where E is the PCR efficiency) [10]:

(2) $$\mathrm{RSD}(\%)=\sqrt{(1+E{)}^{ln(1+E)\times SD(Cq)}-1}$$

**Figure 5:**
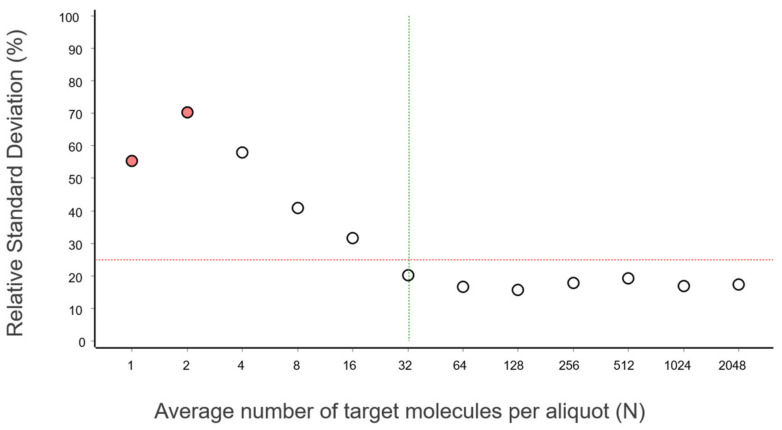
RSD in linear scale expressed in percentage as a function of the expected number of target molecules per calibration sample (N). Red circles indicate that some replicates in those calibrators were negative. A red horizontal dashed line is drawn at 25% RSD, and a green vertical dashed line indicates the lowest concentration with an RSD below 25%, which is the LOQ.

The RSD for the data in Figure 4 is plotted as a function of the number of target molecules per calibrator in Figure 5.

The RSD levels off at 32 molecules and stabilizes, which is consistent with what can be seen in Figure 4. At these concentrations, various sources of imprecision, such as sample handling, pipetting, and measurement error, become significant. Although these data were generated with the rather unique IntelliQube instrument, repeatability is in line with previous reports using standard qPCR instrumentation [14]. 

For bioanalytical methods, we need to estimate important performance parameters. The two most important are the limit of detection (LOD) and LOQ. LOD is the lowest analyte concentration likely to be reliably distinguished from the blank and at which detection is feasible, while LOQ is the limiting concentration at which the analyte cannot only be reliably detected but at which some predefined goals for bias and imprecision are met [15]. The LOQ may be equivalent to the LOD but is usually higher.

For qPCR, there is no single universal recommendation for LOQ. It depends on the assay, sample type, matrix, and purpose. But to be in line with other bioanalytical methods, a maximum RSD of 25% in linear scale is commonly used. Sometimes the RSD increases towards very low and also towards very high concentrations. In those cases, we refer to the lower limit of quantification (LLOQ) and the upper limit of quantification (ULOQ). The LLOQ is then the lowest concentration where RSD ≤ 25%, and the ULOQ is the highest concentration where RSD ≤ 25%.

In Figure 5, we see that 32 target molecules and higher per sample meet this criterion. Thus, the LLOQ of the reference assay, when analyzing purified gDNA standard using the IntelliQube qPCR instrument, is 32 molecules. There is no ULOQ within the concentration range studied.

### 3.3. Sampling Uncertainty

PCR, using an optimized assay, can amplify a single target molecule as routinely demonstrated in dPCR. If we take a cell that contains a single DNA and place it into a qPCR tube so we are sure it is there, and add PCR reagents and an optimized assay for a target sequence in the DNA, we will detect it.

For most samples, however, we do not control the number of target molecules. Consider a 1 mL homogeneous sample containing 1000 lysed cells. From this solution, transfer 1 µL, which is 1/1000th of the volume, to a tube for analysis with qPCR targeting the DNA. The aliquot thus withdrawn can, of course, contain one DNA molecule. However, it may also contain two, perhaps three, or even more DNA molecules. These cases will produce a product, and the PCR will be positive. But the aliquot (pipetted sample aliquot) may contain no DNA, in which case the PCR will be negative. Since the original sample did contain DNA, this is a false negative. 

Stochastic variation in the number of targets across replicates (aliquots) gives rise to sampling uncertainty. Sampling uncertainty becomes important when few targets are expected (small N). 

**Figure 6:**
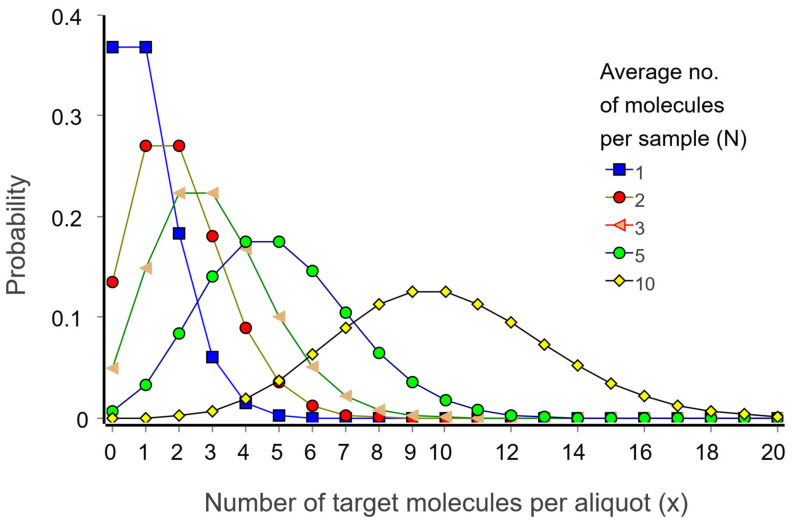
Poisson distributions showing the frequencies of aliquots containing different numbers of target molecules (x) for expectation values: N = 1, 2, 3, 5, and 10 molecules per aliquot.

The probability that an aliquot contains x target molecules, P(x), is given by the Poisson distribution shown in Equation (3):

(3) $$P(x)=\frac{e^{-N}N^{x}}{x!}$$

where N is the expectation value, which is equivalent to the average number of target molecules per aliquot, x is the actual number of targets in a particular aliquot, and ! indicates the factorial of the number (x! = 1 × 2 × 3 × … × x). Figure 6 shows Poisson distributions for selected expectation values. 

Let us inspect the Poisson distributions in Figure 6. For an expectation value of one molecule per aliquot (N = 1), the probability that an aliquot contains one molecule (x = 1) is 37%. Probability is 18% that it contains two target molecules (x = 2); 6% that it contains three (x = 3), and 1.5% that it contains four (x = 4). There is also a relevant 37% probability that an aliquot is negative (x = 0).

**Figure 7:**
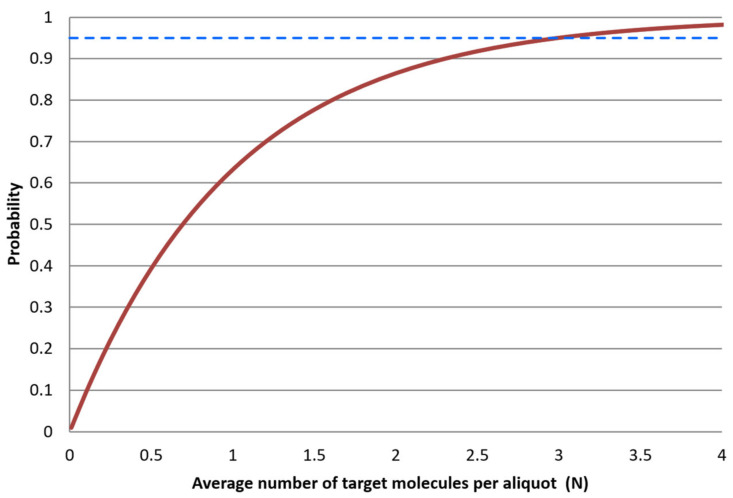
The probability that an aliquot is positive, P(x > 0), as a function of the expected average number of target molecules per aliquot N (red solid line). The blue dashed line is drawn at P(x > 0) = 95%.

Figure 7 shows the probability that an aliquot is positive, P(x > 0), as a function of the average number of target molecules per aliquot (N). 

### 3.4. Limit of Detection (LOD)

When analyzing test samples, we want to be reasonably confident that we obtain a positive test result when the sample is expected to contain even very few target molecules per analyzed volume (N). The challenge here is often to decide when the signal obtained cannot be statistically confounded with the signal from a negative sample, i.e., matrix. While a buffer alone shall not produce a positive PCR, a negative sample may contain similar nucleic acids that amplify. Such a background signal must then be taken into account, limiting the LOD. 

LOD can be limited by many factors related to the sampling and processing of the samples, instrumental noise, quality of reagents, assay performance, etc. Under ideal conditions, when limited by the aliquoting uncertainty, LOD can be predicted. Working at a 95% confidence level means at least 95% of replicates (aliquots) shall be qPCR positive when the sample contains targets. In Figure 7, the dashed line indicates that an average of 3 molecules per analyzed volume (N) is required for an aliquot to be positive in 95% of cases. Hence, the theoretical LOD is N = 3 molecules when aliquoting uncertainty dominates. This theoretical limit is independent of the analytical method.

In the standard curve in Figure 3 and its corresponding residuals plot in Figure 4 we see the spread of replicates increases towards lower concentrations, which is due to increasing aliquoting uncertainty. The effect is visualized and quantified in Figure 5. 

The mean Cq of the replicates at the lowest concentrations (N ≤ 2), where some aliquots are negative, is lower than that predicted by the linear regression (Figure 3 and Figure 4). This is because the average is calculated only for the positive aliquots that have Cq values, which introduces bias. Negative aliquots are also the reason why RSD is lower for N = 1 than for N = 2 (Figure 5). 

**Figure 8:**
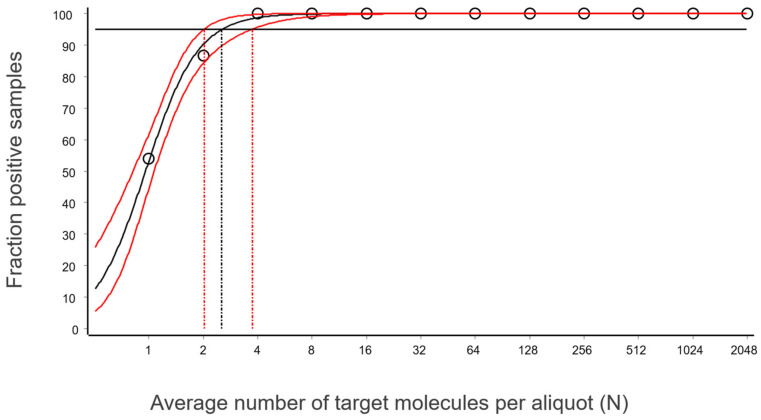
Fraction of aliquots that were positive as a function of N. Data are fitted to a sigmoidal curve (black solid line) to estimate LOD as the concentration that produces 95% positive replicates (LOD = 2.5 molecules, black vertical dashed line). Confidence band of the fit (red lines) is used to estimate the confidence interval (CI) of LOD (CI = [2.0,3.7]).

The fraction of aliquots that were positive as a function of N for the data in Figure 3 is shown in Figure 8. The data are fitted with a sigmoidal function to interpolate the expectation value at 95% probability for aliquots to be positive. The confidence area of the fitted curve is also estimated, from which the confidence range of the LOD is obtained [10].

For the reference assay, targeting purified synthetic DNA measured using the IntelliQube, the estimated LOD at 95% confidence is 2.6 target molecules per aliquot, with a 95% confidence interval (CI) of 2.0–3.7 target molecules. The CI encompasses the theoretical limit of three molecules, and we conclude that the reference assay, when applied to purified synthetic targets, reaches this limit. Switching to dPCR will not improve the sensitivity, since it is limited by the aliquoting uncertainty. To achieve higher sensitivity, larger pipetting volumes should be used, or the sample may be concentrated.

It is worth noting here that the approaches currently used to estimate LOD and LOQ are based on definitions from 1975 and 1980, respectively. These have issues. LOD can be calculated differently depending on the setup, particularly how the ‘blank’ is defined, and suffers from a high false negative rate (often around 50%), and the LOQ threshold, here set at 25%, is arbitrary. In 1993, IUPAC, ISO, and the European Union initiated harmonizing criteria and agreed on new LOD and LOQ definitions that are derived from the straight line fit and error propagation [16,17,18]. This solves the issue of high false rates and the ambiguous LOQ [19]. A general-purpose introduction is found elsewhere [20]. When these new definitions will be introduced into PCR analytics remains to be seen.

The extreme standard curve presented here is based on purified synthetic DNA measured under near-ideal conditions and the LOD and LOQ determined are for the qPCR analysis only. In real biological or clinical samples, matrix-associated inhibitors, nucleic acid degradation, and pre-analytical variability may alter apparent PCR efficiency and shift both LOD and LOQ. Taking into account preanalytical steps, LOD and LOQ will increase, sometimes several orders of magnitude, due to dilutions and volumes of the aliquots. Consequently, the values derived from this illustrative example reflect behavior under controlled conditions. In practice, matrix-specific validation remains essential to establish performance characteristics relevant to the intended sample type.

### 3.5. Expected Imprecision

**Figure 9:**
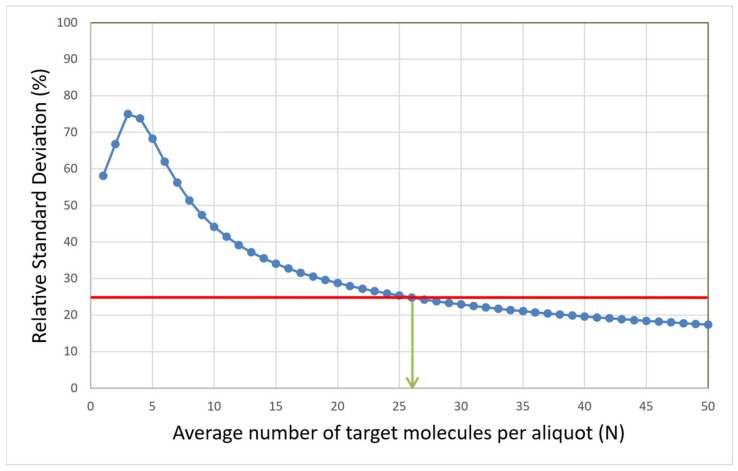
RSD due to aliquoting uncertainty calculated from the Poisson distribution for different expected numbers of target molecules per aliquot (N). The red line is at RSD = 25%. The green arrow indicates the expected number of target molecules (N) at which RSD is 25%, which is 26 molecules. This defines the theoretical LOQ.

With the Poisson model (Equation (3)), the expected imprecision in logarithmic scale due to aliquoting uncertainty expressed as either SD or RSD can be calculated using (Equation (2)). Figure 9 shows the predicted RSD as a function of the expected number of target molecules per aliquot (N). 

From Figure 9, it follows that under conditions when other contributions than aliquoting uncertainty to imprecision are negligible, RSD reaches 25%, which is a common criterion for LOQ, around N = 26 target molecules per aliquot. This is the theoretical LOQ at 25% RSD. 

For the reference assay in Figure 5, RSD drops below 25% at N = 32 target molecules, which is consistent with the theoretical expectations.

## 4. Variance PCR for Absolute Quantification

**Figure 10:**
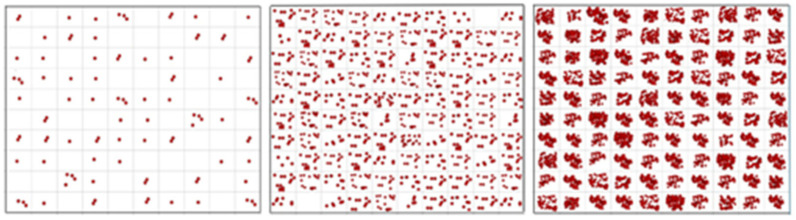
Illustration of expected distributions of target molecules when withdrawing 100 aliquots with N = 1 (**left**), 10 (**middle**), and 100 target (**right**) molecules on average per volume.

Note the resemblance of the theoretical curve in Figure 9 to the experimentally determined RSD of the reference assay in Figure 5. This suggests that when aliquoting, uncertainty dominates variation across aliquots, and the expected number of target molecules per aliquot can be predicted. The concept of this approach, which we term variance PCR (varPCR), is illustrated in Figure 10. This is intended as a conceptual extension to illuminate the fundamental concept of the limit of quantification.

While the targets in the aliquots are clearly observed to vary when the average is one target molecule per aliquot, one hardly notices variation among aliquots at N = 100 molecules on average. Although SD increases with N, the RSD decreases. This dependence was, precisely, presented in Figure 9.

An apparent ambiguity is that the same RSD is obtained at two different expectation values (N), one at each side of the maximum RSD (Figure 9). This, however, is not an issue. At low concentration, the drop in RSD is due to some replicates becoming negative, while at high concentration, all replicates are positive.

A comparison between experimentally measured RSD (Figure 5) and theoretically predicted RSD assuming aliquoting uncertainty only (Figure 9) is provided in Table 1. 

Given that measurements are performed on a logarithmic scale, the agreement between the predicted average number of targets per aliquot derived from the measured RSD and the expected number of targets based on the dilutions is remarkably good. For all samples, the predicted values exceed the expected values somewhat, which may indicate that the expectation values based on dilutions of the stock solution, whose concentration was determined by dPCR, could be slightly underestimated.

At conditions where aliquoting uncertainty dominates, N can be determined directly from the SD (or RSD) of the Cq values of replicate aliquots. This represents an alternative approach to absolute quantification that differs fundamentally from dPCR. 

A potential application of varPCR is as a complement to dPCR, particularly on dPCR platforms capable of real-time fluorescence detection that generate Cq values. At low target loading, where a significant fraction of reaction partitions (which are equivalent to aliquots) are negative, standard dPCR readout provides optimal precision. At higher target loading, when most or all partitions are positive and classical dPCR loses resolving power, target concentration can instead be estimated by the varPCR principle based on the SD of the Cq values. Combining these approaches would dramatically expand the dynamic range of absolute quantification achievable with dPCR platforms.

Although varPCR provides a useful theoretical framework linking Cq variation to absolute target copy number, its reliable implementation would require a substantial number of independent replicate samplings and careful control of non-Poisson sources of variation, including pipetting error, matrix effects, and instrument-related imprecision. Under typical laboratory conditions, these additional sources of variability may dominate over aliquoting uncertainty, limiting the practical applicability of varPCR. We therefore present varPCR primarily as a conceptual extension that clarifies the fundamental constraints of quantification rather than as a routine analytical protocol. However, with the advent of dPCR platforms that read out fluorescence after every cycle, hence combining dPCR/qPCR in one instrument, varPCR analysis can extend the dynamic range as it allows concentrations to be estimated at levels close to and above saturation, where dPCR precision is lost.

## 5. qPCR Standard Curve Under Standard Conditions

**Figure 11:**
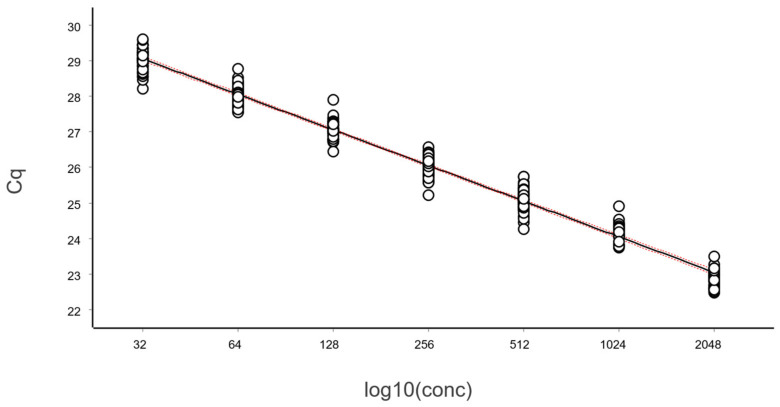
Standard curve based on Cq values vs. N at concentrations above LLOQ. Data fitted to a straight line (black) with linear regression. Uncertainties of the fit are reflected by the Working–Hotelling confidence area indicated with red dashed lines.

**Figure 12:**
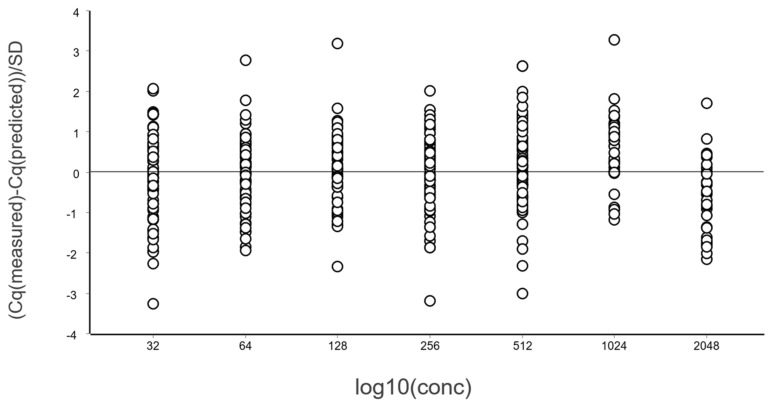
Standardized residuals of the data in Figure 11.

The dynamic range of a qPCR method extends from LLOQ to ULOQ, defined as the concentration range over which the RSD remains below a specified threshold, like 25% in our example. For the reference assay, RSD does not exceed 25% at high concentrations. Figure 11 shows the standard curve above the LLOQ, and Figure 12 shows the corresponding standardized residuals, i.e., residuals scaled by the SD, which is a suitable means for testing for the presence of outliers. Considering each level separately, data points outside ±3 are considered to have an outlying behavior. Alternatively, the traditional Grubbs’ test can be applied to the overall set of residuals, which assumes they follow a normal distribution as required also for the OLS [21].

Within this range, replicate variability is effectively independent of concentration. This is referred to as homoscedasticity in statistics and is an assumption behind linear regression when based on the standard least squares criterion. 

### 5.1. PCR Efficiency

Linear regression of the data in Figure 11 yields the slope and intercept, along with the Working–Hotelling 95% confidence band, shown with red dashed lines. The confidence region is derived from error propagation and, for this example, is exceedingly small, which is a positive and direct consequence of the large number of replicates. The PCR efficiency (E) is estimated as in Equation (4) [6]:

(4) $$E=10^{-k^{-1}}-1$$

The standard error (SE) of the mean of the PCR efficiency can be estimated using Equation (5) [6]:

(5) $$SE\left(E\right)=SE\left(k\right)\times \frac{\left(1+E\right)\ln10}{k^{2}}$$

for which the Student’s 95% CI, considering the t factor at 95% confidence level and n − 2 degrees of freedom, with n being the total number of samples used in the curve, is calculated using Equation (6) [22]:

(6) $$E\pm t_{95\%,n-2}\times SE\left(E\right)$$

For the data in Figure 11, we obtain the following:

- Slope (k): −3.33 [−3.38, −3.28]
- Intercept (m): 34.1 [34.0, 34.2]
- Efficiency (E): 0.997 [0.977, 1.017]

### 5.2. Real-Life Standard Curves

**Figure 13:**
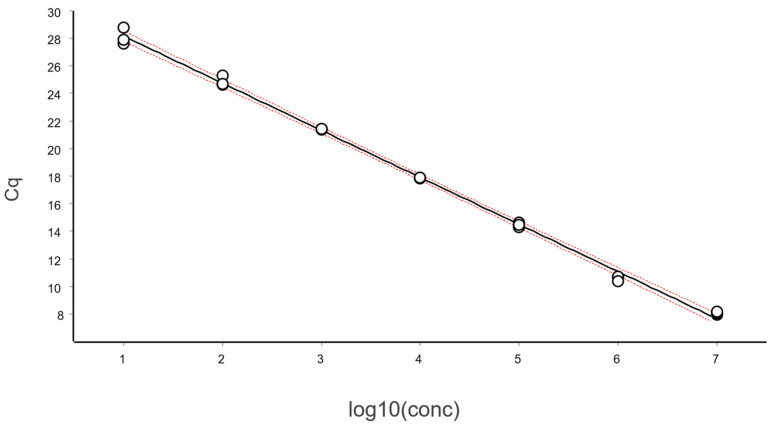
Calibration samples were measured in triplicate in dilution steps of ten, covering six logs in concentration. Data are fitted to the black line using ordinary linear regression. Red dashed lines show the Working–Hotelling confidence area, illustrating the precision of the fit.

Typical standard curves have fewer replicates. However, the calibration samples shall be independent and handled the same way as future test samples, including pre-analytics [13]. To obtain the example in Figure 13, calibration samples were measured in triplicate in dilution steps of ten, covering six logs in concentration. 

The data are fitted using OLS, which yields the following regression parameter estimates along with their 95% CIs:

- Slope (k):−3.42 [−3.50, −3.34]
- Intercept (m): 31.58 [31.21, 31.96]
- Efficiency (E): 0.960 [0.928, 0.992]

**Figure 14:**
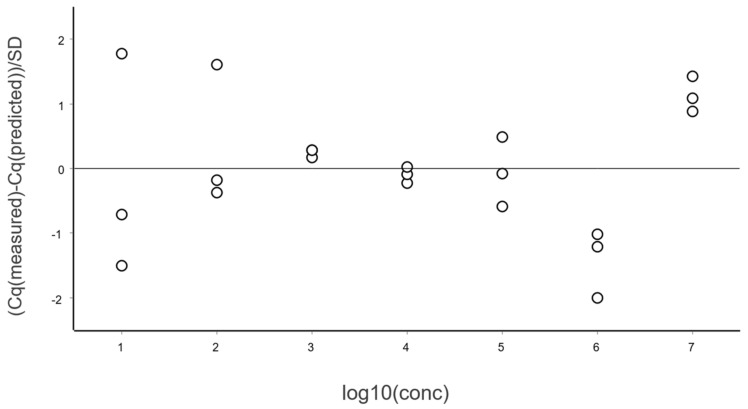
Standardized residuals of the data in Figure 13.

The PCR efficiency, estimated as 96 ± 3%, is well within acceptable limits. Also shown in Figure 13 is the Working–Hotelling area illustrating the precision of the fit. The standardized residuals plot is shown in Figure 14.

### 5.3. Validation of the Linear Dynamic Range

While the dynamic range extends from LLOQ to ULOQ, the linear range, which is usually narrower [23], corresponds to the interval where Cq is proportional to log(N). The linear range (Equation (7)) can be assessed by fitting the data to higher-order polynomial models and testing whether the k_1_ and k_2_ terms in Equations (8) and (9) have statistical significance [13]:

(7) $$y=b_{1}x+m$$

(8) $$y=kx+k_{1}x^{2}+m$$

(9) $$y=kx+k_{1}x^{2}+k_{2}x^{3}+m$$

The second-order polynomial accounts for curvature at one end of the concentration range, while the third-order model takes into account curvature at both ends. An F-test is used to determine whether the inclusion of higher-order terms significantly improves the fit compared with the linear model. We will refer to this as ‘polynomial test’ [24]. 

The full dataset spanning six logs, when fitted by OLS regression, fails the polynomial test for the overall linear dynamic range, i.e., the distribution of the calibrators does not follow a straight line. This result may initially appear surprising, as no other indicators suggest a problem. However, closer inspection of the residuals plot reveals that all three replicates at the highest concentration exhibit positive residuals, while all three replicates at the second-highest concentration exhibit negative residuals. This systematic pattern indicates curvature at the upper end of the range.

**Figure 15:**
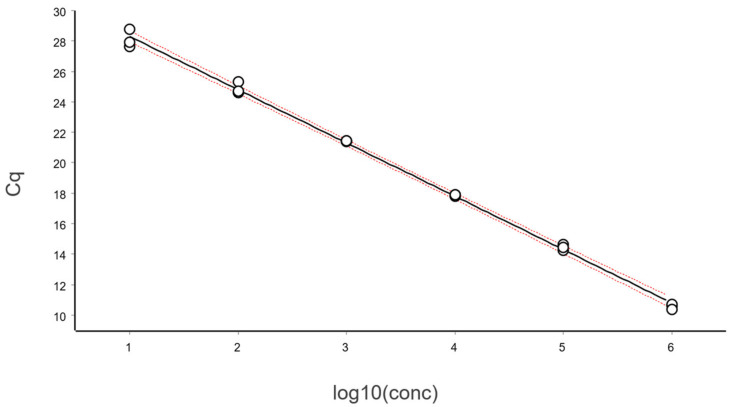
Standard curve using the same data as Figure 13, but leaving out the most concentrated calibration sample, covering only 5 logs of concentration. Working–Hotelling confidence area indicated with red dashed lines.

After removing the three highest-concentration calibration samples, the revised standard curve is shown in Figure 15.

Fit by linear regression yields the following parameters:

- Slope (k): −3.50 [−3.59, −3.41]
- Intercept (m): 31.80 [31.44, 32.16]
- Efficiency (E): 0.930 [0.897, 0.964]

**Figure 16:**
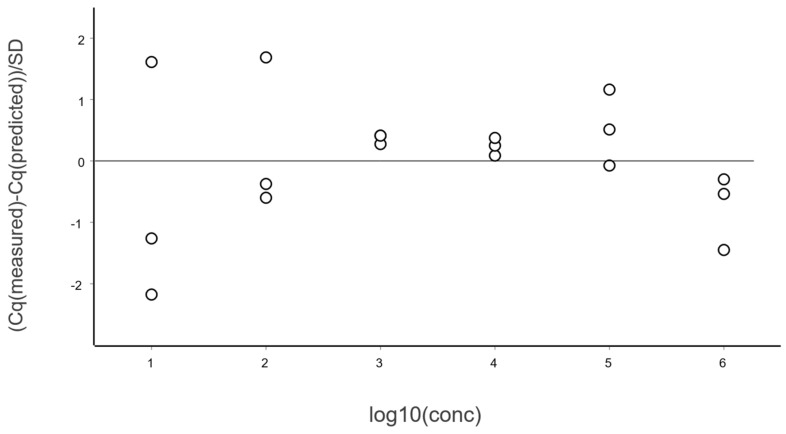
Standardized residuals of the data in Figure 15.

The corresponding standardized residuals plot is shown in Figure 16. 

Although a slight curvature at high concentration may still be observed, it is not statistically significant. The data now pass the polynomial test, and we conclude the method exhibits a linear dynamic range of five orders of magnitude. The PCR efficiency estimate is somewhat reduced to 93 ± 3% but remains within acceptable limits.

The polynomial test is very sensitive to deviations and must be used thoughtfully. When the calibration samples fall very precisely on a straight line, the test may indicate deviations that are purely noise dependent and may suggest removing good data points. We therefore recommend considering also the “allowable deviation from linearity”, as stated elsewhere [13]. If it is below 20%, we would typically accept the standard curve. 

Deviations from linearity at high concentrations can arise from several factors, including inhibition in undiluted samples, errors in baseline subtraction when fluorescence accumulates at very early cycles, or incorrect dilutions. In many cases, the exact cause remains unidentified. If uncorrected, the too high Cq value of the most concentrated sample causes a tilt of the fitted straight line, reducing its slope. A reduced slope implies a higher PCR efficiency, which is incorrect. The impact can be large due to the lever effect and may result in PCR efficiency estimates above 100%.

The true PCR efficiency cannot exceed 100%, which corresponds to copying all molecules in the sample every amplification cycle. An estimate, though, may be higher due to experimental error and imprecision. This is reflected by the CI of the PCR efficiency estimate, which then should encompass 100%.

If the lower confidence range of the PCR efficiency estimate is above 100%, the result should not be accepted as, most likely, concentrations outside the linear dynamic range are included, causing an artificial increase in the efficiency estimate.

### 5.4. Impact of Outliers

**Figure 17:**
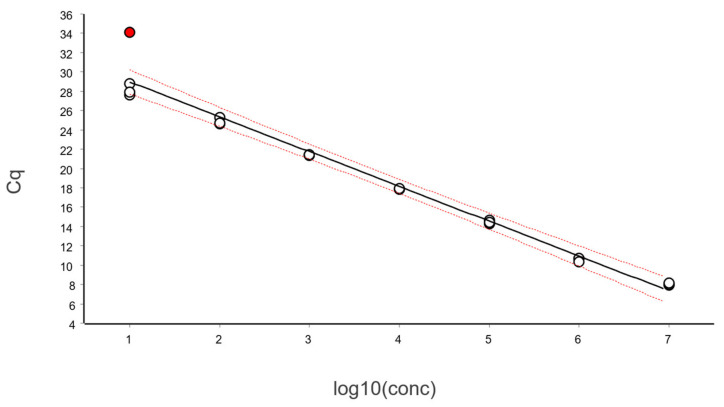
The data in Figure 13 with an outlier sample (red circle) at the lowest concentration. Working–Hotelling confidence area indicated with red dashed lines.

In Figure 17 we have included an outlier sample in the standard curve in Figure 13 for illustrative purposes. 

The outlier is readily identified by visualizing the standardized residuals or with a hypothesis test, such as the Grubbs’ one, and would normally be removed. However, here we keep it to illustrate its impact.

The linear regression yields the following estimates (95% CI):

- Slope (k): −3.61 [−3.88, −3.33 ]
- Intercept (m): 32.57 [31.34, 33.79]
- Efficiency (E): 0.894 [0.800, 0.988]

The estimated PCR efficiency is 89.4%, close to the commonly accepted threshold of 90%. This illustrates that PCR efficiency alone is not a sensitive indicator of problems in standard curve data.

In contrast, the CI of the PCR efficiency is highly informative. Although 89.4% is the point estimate, the 95% confidence range spans from 80% to 99%, which is excessively wide and provides little certainty about the actual PCR efficiency. Indeed, the width of the CI is a more meaningful quality indicator than the point estimate itself.

When applying the polynomial test to study the goodness of the linear fit, the presence of the outlier prevents the detection of the deviation from linearity that was clearly visible in Figure 17. This occurs because the outlier introduces substantial imprecision and tilts the least squares fit to itself. This not only reduces the precision of the estimated PCR efficiency, but also affects the entire linear regression, as reflected by the very broad Working–Hotelling confidence band. Due to this imprecision, the triplicate calibration samples at the second-highest and highest concentrations are no longer significantly below and above the fitted regression line, and the linear range would erroneously be accepted.

This example highlights the critical importance of carefully preparing and assessing the behavior of calibration samples when constructing qPCR standard curves. A single outlier that is not removed can have a profound impact, particularly if it is at an extreme low or high concentration, on regression results and lead to misleading conclusions.

### 5.5. Prediction of Concentrations of Test Samples

**Figure 18:**
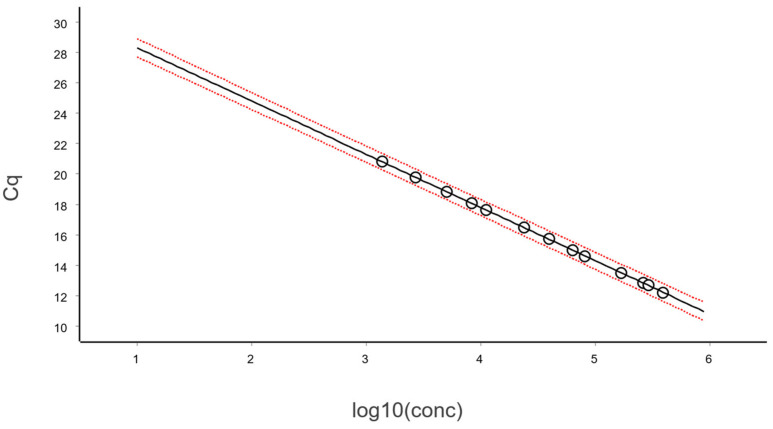
Prediction of the concentrations of test samples using the standard curve established in Figure 15 (black line). Red dashed lines indicate the Working–Hotelling prediction band.

Figure 18 uses the standard curve established in Figure 15 to predict the concentrations of test samples.

For accurate prediction, calibration samples used to construct the standard curve must be handled exactly in the same manner as the test samples and must have exactly the same matrix effects. This ensures that the same sources of confounding variation are maintained. For example, if the test samples were analyzed using several replicates that were subsequently averaged, the calibration samples must be analyzed and averaged in the same way.

Test samples may be available as biological replicates. In such cases, two approaches are possible. The biological replicates can be analyzed separately, yielding independent readouts that may be averaged if a single estimate is desired. Alternatively, the Cq values of the biological replicates can be averaged before estimation, resulting in a single concentration estimate. In the statistical analysis, it is important to account for this averaging, as it reduces variation and therefore improves precision.

The concentration in logarithmic scale of a test sample is estimated by applying Equation (10) and considering the measured Cq and the slope (k) and intercept (m) of the standard curve:

(10) $$log(N)=\frac{Cq-m}{k}$$

The standard curve is typically validated on each sample run using at least two quality control samples at high and low concentrations, with a preference for three (high, medium, and low) concentrations. Intra- and inter-assay accuracy of +/− 1 cycle (corresponding to 50 to 100% relative error (RE) in linear scale) is a common validation criterion [4].

The Working–Hotelling prediction band is wider than the corresponding confidence band for the fit because the former includes three sources of uncertainty. In addition to the imprecision of the slope (k) and the imprecision of the intercept (m), the imprecision of the measured Cq of the test sample also contributes to the imprecision of predictions. 

The standard error, or uncertainty, of the estimated concentration in logarithmic scale of a test sample is given by Equation (11) [25,26].

(11) $$SE_{logN}=\frac{SE_{y.x}}{k}\sqrt{\frac{1}{b}+\frac{1}{n}+\frac{{\left(Cq-\bar{Cq}\right)}^{2}}{k^{2}\sum_{i=1}^{n}{\left(\log N_{i}-\bar{\log N}\right)}^{2}}}$$

The equation looks complex, although it can be implemented readily in a spreadsheet. It is worth looking into the different parameters it contains to understand its essentials. 

$SE_{y.x}$ is the standard error of the least squares fit or the average error of the standard curve. It reflects the average spread of data in the residuals plot in Figure 16. The lower the $SE_{y.x}$, the better the fit of the experimental points to the line.

b is the number of test sample replicates. The more replicates, the higher the precision of the predictions. When measurement error dominates, precision scales with the square root of b.

n is the total number of calibration samples used to construct the standard curve. n = the number of levels × the number of replicates at each level. For method development, a minimum of nine levels is recommended in at least four replicates [13].

${\left(Cq-\bar{Cq}\right)}^{2}$ is the difference between the measured Cq and the average Cq of all the standard samples, squared. Its effect is that precision is highest at the center of the standard curve and decreases towards the edges, as reflected by the Working–Hotelling band. 

$\sum_{i=1}^{n}{\left(\log N_{i}-\bar{\log N}\right)}^{2}$ is the sum of squared differences between the calibration samples’ concentrations and the mean concentration of all the calibration samples, in logarithmic scale. This is the analytical measuring interval of the standard curve and should be within the linear dynamic range. The wider the interval, the greater the precision of the predicted concentrations. 

From the SE of the interpolated value, the CI is obtained considering Equation (12):

(12) $$logN\pm t_{95\%,2tails,n-2}\times SE_{logN}$$

This is on a logarithmic scale, and the CI is symmetric around the point estimate (Table 2).

For example, the estimated concentration of sample 5, with the corresponding 95% CI, is logN_5_ = 4.91 [4.73, 5.09].

Note the CI is symmetrical around the point estimate, and the relative error is about 7.3% (=100 × (5.09 − 4.73)/4.91). In a linear scale, the CI is asymmetric. The best estimate is *N*_5_ = 81,200 [54, 100, 121, 900] molecules, which has an RE of 83.5% (=100 × (121,900 − 54,100)/81,200).

## 6. Conclusions

qPCR standard curves remain a key analytical approach for assay validation and target concentration estimation, but their interpretation depends strongly on whether they are constructed correctly and on the purpose for which they are used. In this paper, we show that standard curves generated with very large numbers of replicates reveal features of qPCR behavior that are not readily apparent under routine conditions, particularly the relationship between replicate variation, detection limits, and target copy number. These features are always present but are usually masked by limited replication.

Standard curves constructed under typical laboratory conditions, with relatively few replicates and independently prepared calibration samples, remain appropriate and informative for routine assay validation and quantification, provided their limitations are recognized. In such cases, careful assessment of dynamic range, outliers, efficiency estimates, and prediction uncertainty is essential for obtaining reliable results.

For clinical diagnostics and the development of standards, the standard curve is essential. Typically, a larger number of replicates is used to enhance precision during method development, while for verification, a smaller number is sufficient. The replicate numbers shall be decided based on the required precision in each case and will depend on sample complexity and how much of the pre-analytics is included. 

We introduce a novel concept termed varPCR to formalize the theoretical relationship between Cq variation and absolute target copy number. This concept is intended to clarify fundamental constraints on quantification rather than to propose a new routine methodology. 

The examples presented here highlight that standard curves are purpose-dependent analytical tools. Understanding what information they can provide, and equally what they cannot, is critical for avoiding common analytical errors and for ensuring robust interpretation of qPCR data.

## Figures and Tables

**Table 1:** Comparison of predicted and measured relative standard deviation (RSD) for the expected number of target molecules below and around LOQ.

Expected Number of Targets (N)	Predicted RSD	Measured RSD	Predicted Number of Targets (N)
1	0.581	0.554	2
2	0.668	0.703	4.5
4	0.738	0.581	6.5
8	0.514	0.408	11.5
16	0.327	0.316	17
32	0.221	0.201	53
64	0.153	0.167	75

**Table 2:** Estimated concentrations with 95% CIs in logarithmic and linear scales for the test samples in Figure 18.

	CI (min)	Log10 (Conc)	CI (max)	CI (min)	Conc.	CI (max)
Test 1	4.44	4.59	4.75	27,500	39,400	56,400
Test 2	3.79	3.92	4.05	6240	8320	11,100
Test 3	3.01	3.14	3.26	1030	1380	1830
Test 4	4.23	4.38	4.52	17,100	23,900	33,300
Test 5	4.73	4.91	5.09	54,100	81,200	122,000
Test 6	3.31	3.43	3.55	2060	2710	3570
Test 7	4.64	4.81	4.98	43,300	63,900	94,500
Test 8	5.25	5.46	5.68	176,000	290,000	478,000
Test 9	3.92	4.04	4.17	8240	11,100	14,900
Test 10	5.03	5.23	5.43	107,000	169,000	267,000
Test 11	3.58	3.71	3.82	3800	5020	6620
Test 12	5.21	5.42	5.63	161,000	264,000	431,000
Test 13	5.37	5.59	5.82	233,000	393,000	663,000

## Data Availability

The data used in the examples, including guidance, are available at https://www.multid.se/qpcrstandardcurve/ (accessed on 14 March 2026).

## Abbreviations

The following abbreviations are used in this manuscript:

b	number of test sample replicates
cDNA	complementary DNA
CI	confidence interval
Cq	quantification cycle
dPCR	digital PCR
E	PCR efficiency
gDNA	genomic DNA
ISO	International Organization for Standardization
IUPAC	International Union of Pure and Applied Chemistry
k	slope of the standard curve
LLOQ	lower limit of quantification
LOD	limit of detection
LOQ	limit of quantification
m	intercept of the standard curve
MIQE	Minimum Information for Publication of Quantitative Real-Time PCR Experiments
n	total number of standard samples
N	expected (and average) number of target molecules per analyzed volume.
OLS	ordinary least squares regression
P(x)	the probability that an aliquot contains exactly x target molecules
P(x>0)	the probability an aliquot is positive
qPCR	quantitative PCR
RSD	relative standard deviation
SD	standard deviation
SE	standard error
${SE}_{y,x}$	average standard error of the regression
$t_{95\%,n-2}$	the critical value from the Student’s t-distribution required to calculate a 95% confidence interval when the degrees of freedom are n−2
ULOQ	upper limit of quantification
varPCR	variance PCR
x	the actual number of target molecules in an aliquot
